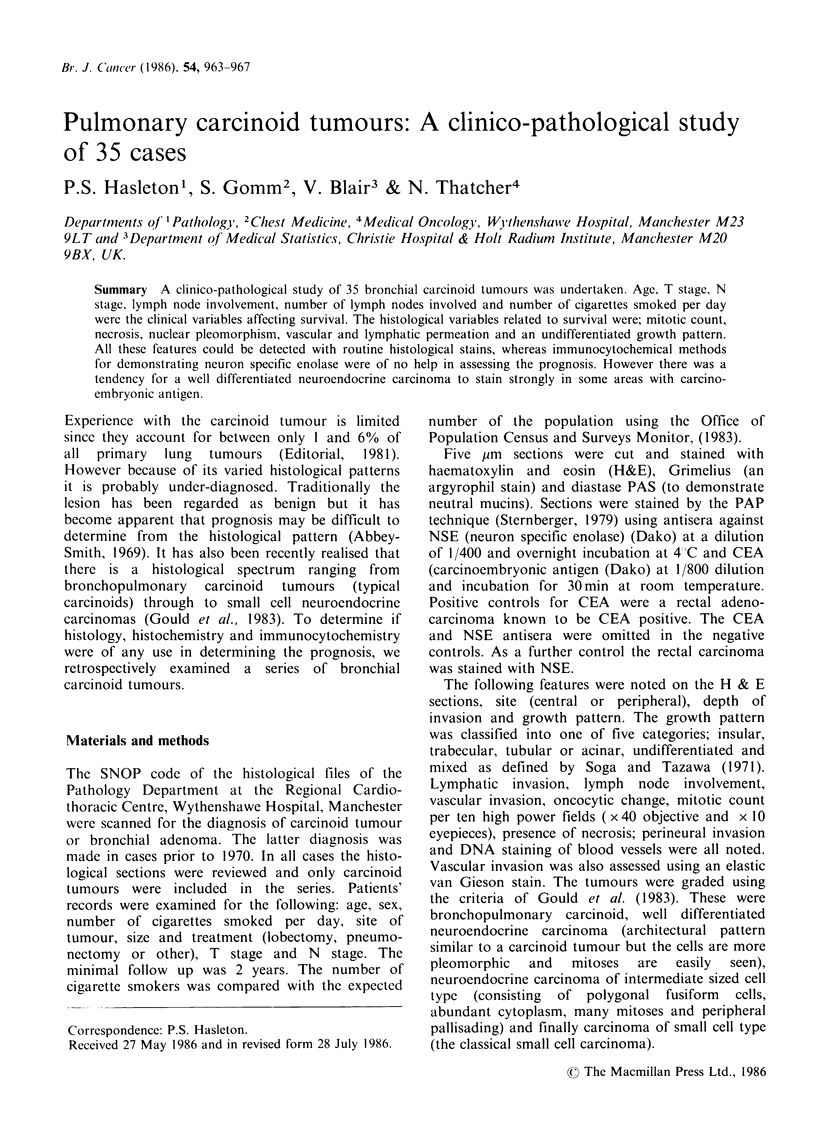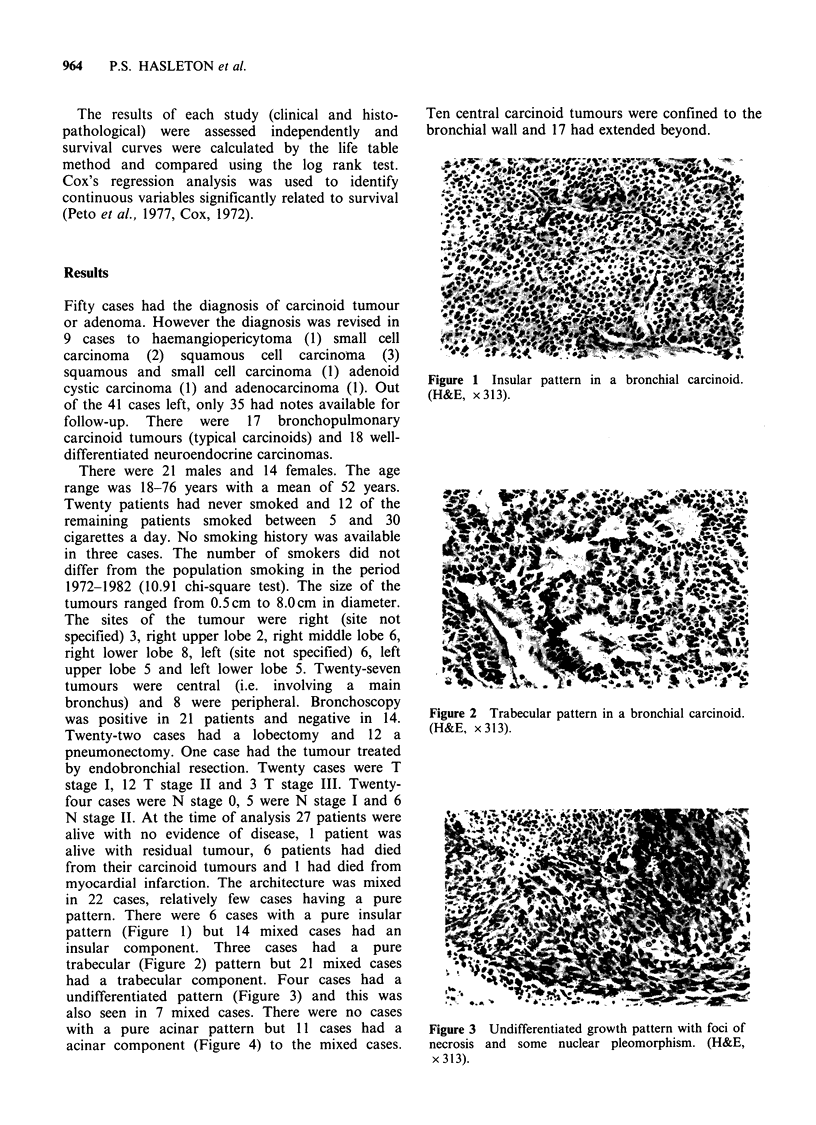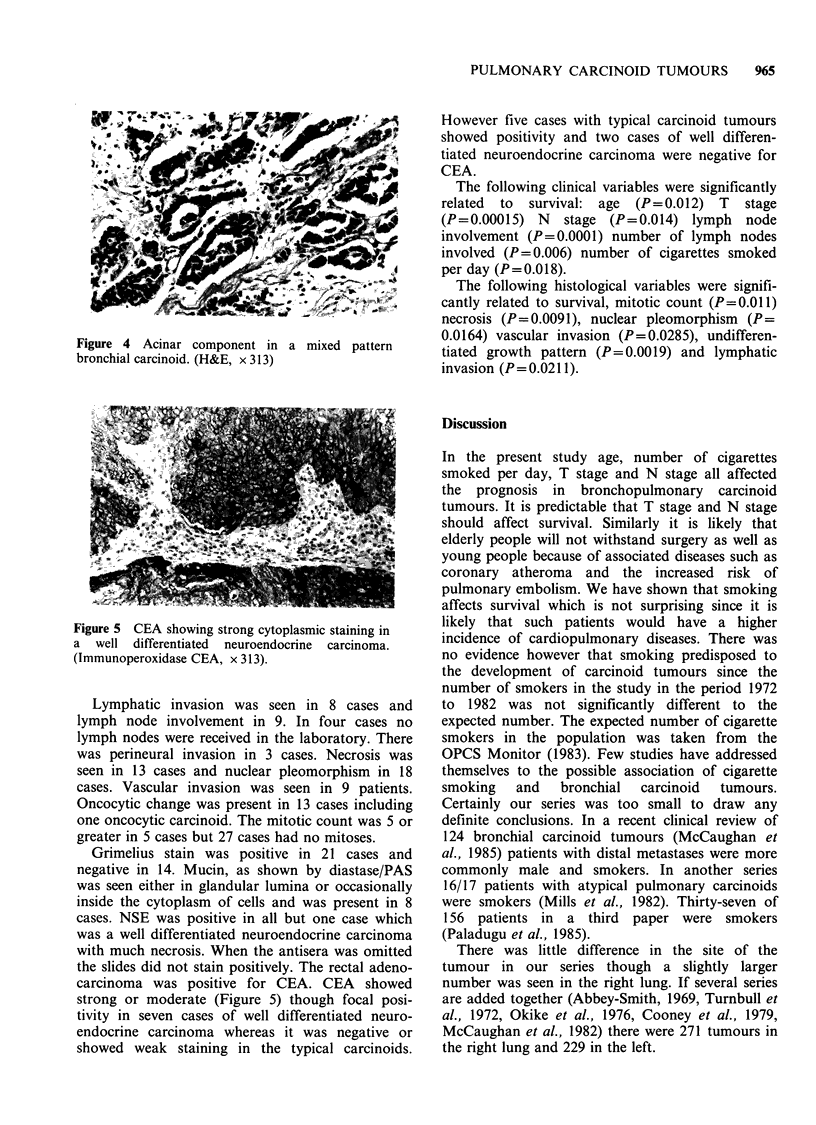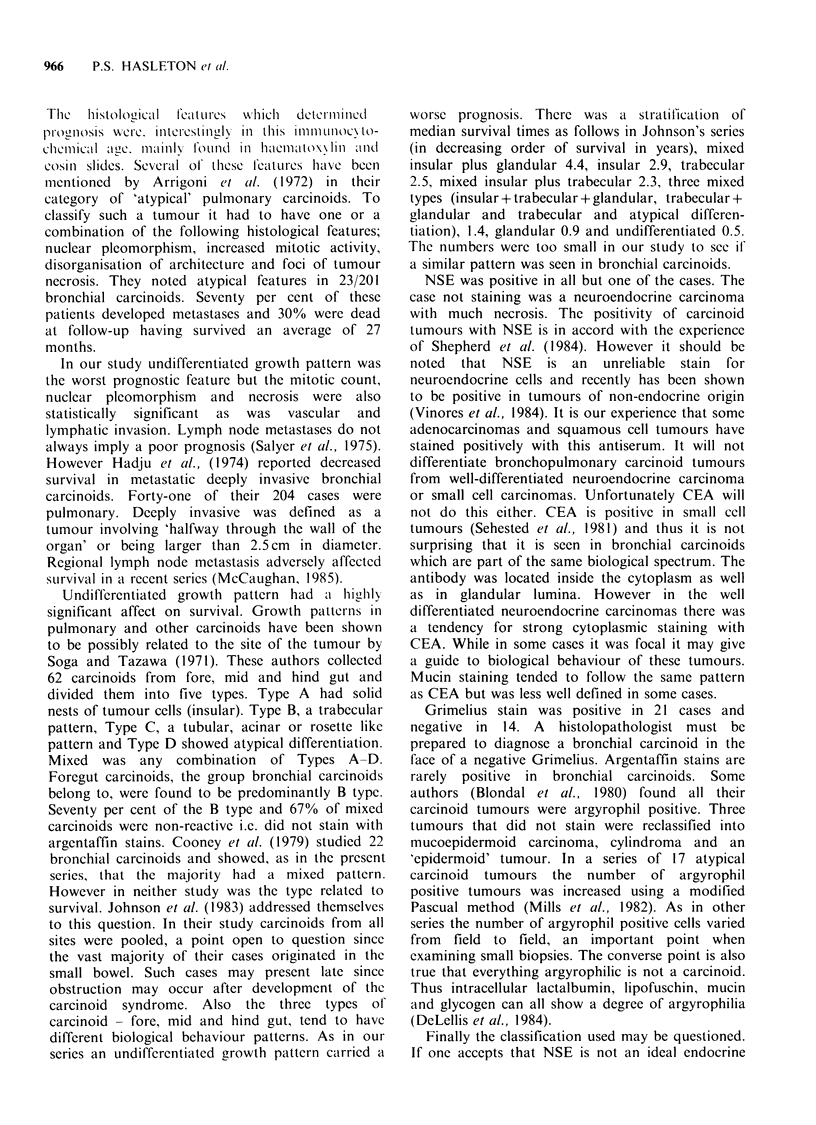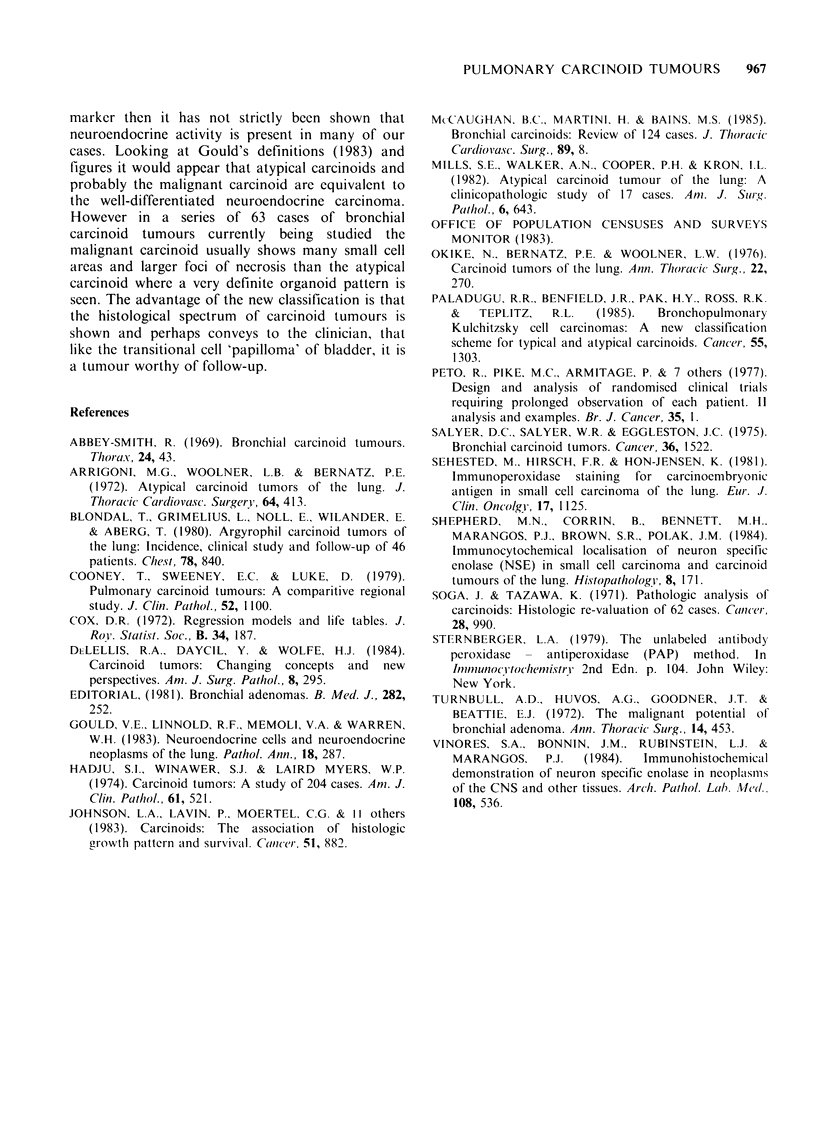# Pulmonary carcinoid tumours: a clinico-pathological study of 35 cases.

**DOI:** 10.1038/bjc.1986.268

**Published:** 1986-12

**Authors:** P. S. Hasleton, S. Gomm, V. Blair, N. Thatcher

## Abstract

**Images:**


*Br. J. Cancer* (1986), **54**, 963–967

### Pulmonary carcinoid tumours: A clinico-pathological study of 35 cases

P.S. Hasleton[1], S. Gomm[2], V. Blair[3] & N. Thatcher[4]

*Departments of [1]Pathology, [2]Chest Medicine, [4]Medical Oncology, Wythenshawe Hospital, Manchester M23 9LT and [3]Department of Medical Statistics, Christie Hospital & Holt Radium Institute, Manchester M20 9BX, UK.*

**Summary** A clinico-pathological study of 35 bronchial carcinoid tumours was undertaken. Age, T stage, N stage, lymph node involvement, number of lymph nodes involved and number of cigarettes smoked per day were the clinical variables affecting survival. The histological variables related to survival were; mitotic count, necrosis, nuclear pleomorphism, vascular and lymphatic permeation and an undifferentiated growth pattern. All these features could be detected with routine histological stains, whereas immunocytochemical methods for demonstrating neuron specific enolase were of no help in assessing the prognosis. However there was a tendency for a well differentiated neuroendocrine carcinoma to stain strongly in some areas with carcino-embryonic antigen.

Experience with the carcinoid tumour is limited since they account for between only 1 and 6% of all primary lung tumours (Editorial, 1981). However because of its varied histological patterns it is probably under-diagnosed. Traditionally the lesion has been regarded as benign but it has become apparent that prognosis may be difficult to determine from the histological pattern (Abbey-Smith, 1969). It has also been recently realised that there is a histological spectrum ranging from bronchopulmonary carcinoid tumours (typical carcinoids) through to small cell neuroendocrine carcinomas (Gould *et al.*, 1983). To determine if histology, histochemistry and immunocytochemistry were of any use in determining the prognosis, we retrospectively examined a series of bronchial carcinoid tumours.

### Materials and methods

The SNOP code of the histological files of the Pathology Department at the Regional Cardio-thoracic Centre, Wythenshawe Hospital, Manchester were scanned for the diagnosis of carcinoid tumour or bronchial adenoma. The latter diagnosis was made in cases prior to 1970. In all cases the histological sections were reviewed and only carcinoid tumours were included in the series. Patients' records were examined for the following: age, sex, number of cigarettes smoked per day, site of tumour, size and treatment (lobectomy, pneumonectomy or other), T stage and N stage. The minimal follow up was 2 years. The number of cigarette smokers was compared with the expected number of the population using the Office of Population Census and Surveys Monitor, (1983).

Five $\mu$m sections were cut and stained with haematoxylin and eosin (H&E), Grimelius (an argyrophil stain) and diastase PAS (to demonstrate neutral mucins). Sections were stained by the PAP technique (Sternberger, 1979) using antisera against NSE (neuron specific enolase) (Dako) at a dilution of 1/400 and overnight incubation at 4°C and CEA (carcinoembryonic antigen (Dako) at 1/800 dilution and incubation for 30 min at room temperature. Positive controls for CEA were a rectal adenocarcinoma known to be CEA positive. The CEA and NSE antisera were omitted in the negative controls. As a further control the rectal carcinoma was stained with NSE.

The following features were noted on the H & E sections, site (central or peripheral), depth of invasion and growth pattern. The growth pattern was classified into one of five categories; insular, trabecular, tubular or acinar, undifferentiated and mixed as defined by Soga and Tazawa (1971). Lymphatic invasion, lymph node involvement, vascular invasion, oncocytic change, mitotic count per ten high power fields ($\times 40$ objective and $\times 10$ eyepieces), presence of necrosis; perineural invasion and DNA staining of blood vessels were all noted. Vascular invasion was also assessed using an elastic van Gieson stain. The tumours were graded using the criteria of Gould *et al.* (1983). These were bronchopulmonary carcinoid, well differentiated neuroendocrine carcinoma (architectural pattern similar to a carcinoid tumour but the cells are more pleomorphic and mitoses are easily seen), neuroendocrine carcinoma of intermediate sized cell type (consisting of polygonal fusiform cells, abundant cytoplasm, many mitoses and peripheral pallisading) and finally carcinoma of small cell type (the classical small cell carcinoma).

Correspondence: P.S. Hasleton.
Received 27 May 1986 and in revised form 28 July 1986.

© The Macmillan Press Ltd., 1986

The results of each study (clinical and histopathological) were assessed independently and survival curves were calculated by the life table method and compared using the log rank test. Cox's regression analysis was used to identify continuous variables significantly related to survival (Peto *et al.*, 1977, Cox, 1972).

### Results

Fifty cases had the diagnosis of carcinoid tumour or adenoma. However the diagnosis was revised in 9 cases to haemangiopericytoma (1) small cell carcinoma (2) squamous cell carcinoma (3) squamous and small cell carcinoma (1) adenoid cystic carcinoma (1) and adenocarcinoma (1). Out of the 41 cases left, only 35 had notes available for follow-up. There were 17 bronchopulmonary carcinoid tumours (typical carcinoids) and 18 well-differentiated neuroendocrine carcinomas.

There were 21 males and 14 females. The age range was 18–76 years with a mean of 52 years. Twenty patients had never smoked and 12 of the remaining patients smoked between 5 and 30 cigarettes a day. No smoking history was available in three cases. The number of smokers did not differ from the population smoking in the period 1972–1982 (10.91 chi-square test). The size of the tumours ranged from 0.5 cm to 8.0 cm in diameter. The sites of the tumour were right (site not specified) 3, right upper lobe 2, right middle lobe 6, right lower lobe 8, left (site not specified) 6, left upper lobe 5 and left lower lobe 5. Twenty-seven tumours were central (i.e. involving a main bronchus) and 8 were peripheral. Bronchoscopy was positive in 21 patients and negative in 14. Twenty-two cases had a lobectomy and 12 a pneumonectomy. One case had the tumour treated by endobronchial resection. Twenty cases were T stage I, 12 T stage II and 3 T stage III. Twenty-four cases were N stage 0, 5 were N stage I and 6 N stage II. At the time of analysis 27 patients were alive with no evidence of disease, 1 patient was alive with residual tumour, 6 patients had died from their carcinoid tumours and 1 had died from myocardial infarction. The architecture was mixed in 22 cases, relatively few cases having a pure pattern. There were 6 cases with a pure insular pattern (Figure 1) but 14 mixed cases had an insular component. Three cases had a pure trabecular (Figure 2) pattern but 21 mixed cases had a trabecular component. Four cases had a undifferentiated pattern (Figure 3) and this was also seen in 7 mixed cases. There were no cases with a pure acinar pattern but 11 cases had a acinar component (Figure 4) to the mixed cases. Ten central carcinoid tumours were confined to the bronchial wall and 17 had extended beyond.

**Figure 1** Insular pattern in a bronchial carcinoid. (H&E, ×313).

**Figure 2** Trabecular pattern in a bronchial carcinoid. (H&E, ×313).

**Figure 3** Undifferentiated growth pattern with foci of necrosis and some nuclear pleomorphism. (H&E, ×313).

**Figure 4** Acinar component in a mixed pattern bronchial carcinoid. (H&E, ×313)

**Figure 5** CEA showing strong cytoplasmic staining in a well differentiated neuroendocrine carcinoma. (Immunoperoxidase CEA, ×313).

Lymphatic invasion was seen in 8 cases and lymph node involvement in 9. In four cases no lymph nodes were received in the laboratory. There was perineural invasion in 3 cases. Necrosis was seen in 13 cases and nuclear pleomorphism in 18 cases. Vascular invasion was seen in 9 patients. Oncocytic change was present in 13 cases including one oncocytic carcinoid. The mitotic count was 5 or greater in 5 cases but 27 cases had no mitoses.

Grimelius stain was positive in 21 cases and negative in 14. Mucin, as shown by diastase/PAS was seen either in glandular lumina or occasionally inside the cytoplasm of cells and was present in 8 cases. NSE was positive in all but one case which was a well differentiated neuroendocrine carcinoma with much necrosis. When the antisera was omitted the slides did not stain positively. The rectal adenocarcinoma was positive for CEA. CEA showed strong or moderate (Figure 5) though focal positivity in seven cases of well differentiated neuroendocrine carcinoma whereas it was negative or showed weak staining in the typical carcinoids. However five cases with typical carcinoid tumours showed positivity and two cases of well differentiated neuroendocrine carcinoma were negative for CEA.

The following clinical variables were significantly related to survival: age ($P=0.012$) T stage ($P=0.00015$) N stage ($P=0.014$) lymph node involvement ($P=0.0001$) number of lymph nodes involved ($P=0.006$) number of cigarettes smoked per day ($P=0.018$).

The following histological variables were significantly related to survival, mitotic count ($P=0.011$) necrosis ($P=0.0091$), nuclear pleomorphism ($P=0.0164$) vascular invasion ($P=0.0285$), undifferentiated growth pattern ($P=0.0019$) and lymphatic invasion ($P=0.0211$).

### Discussion

In the present study age, number of cigarettes smoked per day, T stage and N stage all affected the prognosis in bronchopulmonary carcinoid tumours. It is predictable that T stage and N stage should affect survival. Similarly it is likely that elderly people will not withstand surgery as well as young people because of associated diseases such as coronary atheroma and the increased risk of pulmonary embolism. We have shown that smoking affects survival which is not surprising since it is likely that such patients would have a higher incidence of cardiopulmonary diseases. There was no evidence however that smoking predisposed to the development of carcinoid tumours since the number of smokers in the study in the period 1972 to 1982 was not significantly different to the expected number. The expected number of cigarette smokers in the population was taken from the OPCS Monitor (1983). Few studies have addressed themselves to the possible association of cigarette smoking and bronchial carcinoid tumours. Certainly our series was too small to draw any definite conclusions. In a recent clinical review of 124 bronchial carcinoid tumours (McCaughan *et al.*, 1985) patients with distal metastases were more commonly male and smokers. In another series 16/17 patients with atypical pulmonary carcinoids were smokers (Mills *et al.*, 1982). Thirty-seven of 156 patients in a third paper were smokers (Paladugu *et al.*, 1985).

There was little difference in the site of the tumour in our series though a slightly larger number was seen in the right lung. If several series are added together (Abbey-Smith, 1969, Turnbull *et al.*, 1972, Okike *et al.*, 1976, Cooney *et al.*, 1979, McCaughan *et al.*, 1982) there were 271 tumours in the right lung and 229 in the left.

The histological features which determined prognosis were, interestingly in this immunocytochemical age, mainly found in haematoxylin and eosin slides. Several of these features have been mentioned by Arrigoni *et al.* (1972) in their category of 'atypical' pulmonary carcinoids. To classify such a tumour it had to have one or a combination of the following histological features; nuclear pleomorphism, increased mitotic activity, disorganisation of architecture and foci of tumour necrosis. They noted atypical features in 23/201 bronchial carcinoids. Seventy per cent of these patients developed metastases and 30% were dead at follow-up having survived an average of 27 months.

In our study undifferentiated growth pattern was the worst prognostic feature but the mitotic count, nuclear pleomorphism and necrosis were also statistically significant as was vascular and lymphatic invasion. Lymph node metastases do not always imply a poor prognosis (Salyer *et al.*, 1975). However Hadju *et al.*, (1974) reported decreased survival in metastatic deeply invasive bronchial carcinoids. Forty-one of their 204 cases were pulmonary. Deeply invasive was defined as a tumour involving 'halfway through the wall of the organ' or being larger than 2.5 cm in diameter. Regional lymph node metastasis adversely affected survival in a recent series (McCaughan, 1985).

Undifferentiated growth pattern had a highly significant affect on survival. Growth patterns in pulmonary and other carcinoids have been shown to be possibly related to the site of the tumour by Soga and Tazawa (1971). These authors collected 62 carcinoids from fore, mid and hind gut and divided them into five types. Type A had solid nests of tumour cells (insular). Type B, a trabecular pattern, Type C, a tubular, acinar or rosette like pattern and Type D showed atypical differentiation. Mixed was any combination of Types A–D. Foregut carcinoids, the group bronchial carcinoids belong to, were found to be predominantly B type. Seventy per cent of the B type and 67% of mixed carcinoids were non-reactive i.e. did not stain with argentaffin stains. Cooney *et al.* (1979) studied 22 bronchial carcinoids and showed, as in the present series, that the majority had a mixed pattern. However in neither study was the type related to survival. Johnson *et al.* (1983) addressed themselves to this question. In their study carcinoids from all sites were pooled, a point open to question since the vast majority of their cases originated in the small bowel. Such cases may present late since obstruction may occur after development of the carcinoid syndrome. Also the three types of carcinoid – fore, mid and hind gut, tend to have different biological behaviour patterns. As in our series an undifferentiated growth pattern carried a worse prognosis. There was a stratification of median survival times as follows in Johnson's series (in decreasing order of survival in years), mixed insular plus glandular 4.4, insular 2.9, trabecular 2.5, mixed insular plus trabecular 2.3, three mixed types (insular + trabecular + glandular, trabecular + glandular and trabecular and atypical differentiation), 1.4, glandular 0.9 and undifferentiated 0.5. The numbers were too small in our study to see if a similar pattern was seen in bronchial carcinoids.

NSE was positive in all but one of the cases. The case not staining was a neuroendocrine carcinoma with much necrosis. The positivity of carcinoid tumours with NSE is in accord with the experience of Shepherd *et al.* (1984). However it should be noted that NSE is an unreliable stain for neuroendocrine cells and recently has been shown to be positive in tumours of non-endocrine origin (Vinores *et al.*, 1984). It is our experience that some adenocarcinomas and squamous cell tumours have stained positively with this antiserum. It will not differentiate bronchopulmonary carcinoid tumours from well-differentiated neuroendocrine carcinoma or small cell carcinomas. Unfortunately CEA will not do this either. CEA is positive in small cell tumours (Sehested *et al.*, 1981) and thus it is not surprising that it is seen in bronchial carcinoids which are part of the same biological spectrum. The antibody was located inside the cytoplasm as well as in glandular lumina. However in the well differentiated neuroendocrine carcinomas there was a tendency for strong cytoplasmic staining with CEA. While in some cases it was focal it may give a guide to biological behaviour of these tumours. Mucin staining tended to follow the same pattern as CEA but was less well defined in some cases.

Grimelius stain was positive in 21 cases and negative in 14. A histolopathologist must be prepared to diagnose a bronchial carcinoid in the face of a negative Grimelius. Argentaffin stains are rarely positive in bronchial carcinoids. Some authors (Blondal *et al.*, 1980) found all their carcinoid tumours were argyrophil positive. Three tumours that did not stain were reclassified into mucoepidermoid carcinoma, cylindroma and an 'epidermoid' tumour. In a series of 17 atypical carcinoid tumours the number of argyrophil positive tumours was increased using a modified Pascual method (Mills *et al.*, 1982). As in other series the number of argyrophil positive cells varied from field to field, an important point when examining small biopsies. The converse point is also true that everything argyrophilic is not a carcinoid. Thus intracellular lactalbumin, lipofuschin, mucin and glycogen can all show a degree of argyrophilia (DeLellis *et al.*, 1984).

Finally the classification used may be questioned. If one accepts that NSE is not an ideal endocrine marker then it has not strictly been shown that neuroendocrine activity is present in many of our cases. Looking at Gould's definitions (1983) and figures it would appear that atypical carcinoids and probably the malignant carcinoid are equivalent to the well-differentiated neuroendocrine carcinoma. However in a series of 63 cases of bronchial carcinoid tumours currently being studied the malignant carcinoid usually shows many small cell areas and larger foci of necrosis than the atypical carcinoid where a very definite organoid pattern is seen. The advantage of the new classification is that the histological spectrum of carcinoid tumours is shown and perhaps conveys to the clinician, that like the transitional cell 'papilloma' of bladder, it is a tumour worthy of follow-up.

### References

ABBEY-SMITH, R. (1969). Bronchial carcinoid tumours. *Thorax*, **24**, 43.

ARRIGONI, M.G., WOOLNER, L.B. & BERNATZ, P.E. (1972). Atypical carcinoid tumors of the lung. *J. Thoracic Cardiovasc. Surgery*, **64**, 413.

BLONDAL, T., GRIMELIUS, L., NOLL, E., WILANDER, E. & ABERG, T. (1980). Argyrophil carcinoid tumors of the lung: Incidence, clinical study and follow-up of 46 patients. *Chest*, **78**, 840.

COONEY, T., SWEENEY, E.C. & LUKE, D. (1979). Pulmonary carcinoid tumours: A comparitive regional study. *J. Clin. Pathol.*, **52**, 1100.

COX, D.R. (1972). Regression models and life tables. *J. Roy. Statist. Soc.*, **B. 34**, 187.

DELELLIS, R.A., DAYCIL, Y. & WOLFE, H.J. (1984). Carcinoid tumors: Changing concepts and new perspectives. *Am. J. Surg. Pathol.*, **8**, 295.

EDITORIAL, (1981). Bronchial adenomas. *B. Med. J.*, **282**, 252.

GOULD, V.E., LINNOLD, R.F., MEMOLI, V.A. & WARREN, W.H. (1983). Neuroendocrine cells and neuroendocrine neoplasms of the lung. *Pathol. Ann.*, **18**, 287.

HADJU, S.I., WINAWER, S.J. & LAIRD MYERS, W.P. (1974). Carcinoid tumors: A study of 204 cases. *Am. J. Clin. Pathol.*, **61**, 521.

JOHNSON, L.A., LAVIN, P., MOERTEL, C.G. & 11 others (1983). Carcinoids: The association of histologic growth pattern and survival. *Cancer*, **51**, 882.

McCAUGHAN, B.C., MARTINI, H. & BAINS, M.S. (1985). Bronchial carcinoids: Review of 124 cases. *J. Thoracic Cardiovasc. Surg.*, **89**, 8.

MILLS, S.E., WALKER, A.N., COOPER, P.H. & KRON, I.L. (1982). Atypical carcinoid tumour of the lung: A clinicopathologic study of 17 cases. *Am. J. Surg. Pathol.*, **6**, 643.

OFFICE OF POPULATION CENSUSES AND SURVEYS MONITOR (1983).

OKIKE, N., BERNATZ, P.E. & WOOLNER, L.W. (1976). Carcinoid tumors of the lung. *Ann. Thoracic Surg.*, **22**, 270.

PALADUGU, R.R., BENFIELD, J.R., PAK, H.Y., ROSS, R.K. & TEPLITZ, R.L. (1985). Bronchopulmonary Kulchitzsky cell carcinomas: A new classification scheme for typical and atypical carcinoids. *Cancer*, **55**, 1303.

PETO, R., PIKE, M.C., ARMITAGE, P. & 7 others (1977). Design and analysis of randomised clinical trials requiring prolonged observation of each patient. II analysis and examples. *Br. J. Cancer*, **35**, 1.

SALYER, D.C., SALYER, W.R. & EGGLESTON, J.C. (1975). Bronchial carcinoid tumors. *Cancer*, **36**, 1522.

SEHESTED, M., HIRSCH, F.R. & HON-JENSEN, K. (1981). Immunoperoxidase staining for carcinoembryonic antigen in small cell carcinoma of the lung. *Eur. J. Clin. Oncolgy*, **17**, 1125.

SHEPHERD, M.N., CORRIN, B., BENNETT, M.H., MARANGOS, P.J., BROWN, S.R., POLAK, J.M. (1984). Immunocytochemical localisation of neuron specific enolase (NSE) in small cell carcinoma and carcinoid tumours of the lung. *Histopathology*, **8**, 171.

SOGA, J. & TAZAWA, K. (1971). Pathologic analysis of carcinoids: Histologic re-valuation of 62 cases. *Cancer*, **28**, 990.

STERNBERGER, L.A. (1979). The unlabeled antibody peroxidase – antiperoxidase (PAP) method. In *Immunocytochemistry* 2nd Edn. p. 104. John Wiley: New York.

TURNBULL, A.D., HUVOS, A.G., GOODNER, J.T. & BEATTIE, E.J. (1972). The malignant potential of bronchial adenoma. *Ann. Thoracic Surg.*, **14**, 453.

VINORES, S.A., BONNIN, J.M., RUBINSTEIN, L.J. & MARANGOS, P.J. (1984). Immunohistochemical demonstration of neuron specific enolase in neoplasms of the CNS and other tissues. *Arch. Pathol. Lab. Med.*, **108**, 536.